# Malignant Pleural Mesothelioma with Marked Lymphatic Involvement: A Report of Two Autopsy Cases

**DOI:** 10.1155/2017/6195898

**Published:** 2017-05-29

**Authors:** Reiko Ideguchi, Kazuto Ashizawa, Saori Akashi, Michiko Shindo, Kazunori Minami, Toshio Fukuda, Junji Irie, Minoru Fukuda, Masataka Uetani

**Affiliations:** ^1^Department of Radiology, Nagasaki Harbor Medical Center City Hospital, Nagasaki, Japan; ^2^Department of Clinical Oncology, Nagasaki University Graduate School of Biomedical Sciences, Nagasaki, Japan; ^3^Department of Pathology, Nagasaki Harbor Medical Center City Hospital, Nagasaki, Japan; ^4^Department of Internal Medicine, Nagasaki Harbor Medical Center City Hospital, Nagasaki, Japan; ^5^Department of Radiological Sciences, Nagasaki University Graduate School of Biomedical Sciences, Nagasaki, Japan

## Abstract

We herein report two cases of malignant pleural mesothelioma with marked lymphangiosis. The patients included a 68-year-old man and a 67-year-old man who both had a history of exposure to asbestos. Computed tomography (CT) on admission showed pleural effusion with pleural thickening. In both cases, a histopathological examination of the pleura confirmed the diagnosis of epithelioid malignant mesothelioma. They received chemotherapy, but the treatment was only palliative. The chest CT assessments during admission revealed marked pleural effusion and mediastinal lymphadenopathy. CT also showed a consolidative mass with bronchovascular bundle and septal thickening in the lungs suggesting pulmonary parenchymal involvement and the lymphangitic spread of the tumor. These CT findings mimicked lung cancer with pleuritis and lymphangitic carcinomatosis. Autopsy was performed in both cases. Macroscopically, the tumor cells infiltrated the lung with the marked lymphatic spread of the tumor. Microscopy also revealed that the tumor had invaded the pulmonary parenchyma with the marked lymphatic spread of the tumor. Although this growth pattern is unusual, malignant pleural mesothelioma should be considered as the differential diagnosis, especially in patients with pleural lesions.

## 1. Introduction 

Malignant mesothelioma is a rare neoplasm that arises from the pleura or, rarely, the pericardium or peritoneum [1]. The prognosis is poor due to the diffuse nature of the tumor, with the involvement of the surrounding structures and local progression of the disease generally leading to death within 9–17 months after diagnosis [2]. Extrapleural metastases are present at autopsy in 87.7% of cases [3]. Major sites for metastases are regional lymph nodes, lung, adrenal glands, liver, and kidneys.

Lymphatic spread is known to occur as a microscopic phenomenon. Nind et al. reported that lymphatic spread was seen in 27 cases (13.5%) among a series of 200 patients with malignant pleural mesothelioma pathologically [4], while lymphatic spread is rare even in advanced cases clinically. To the best of our knowledge, the bilaterally diffuse lymphatic spread of malignant pleural mesothelioma has been reported as the predominant radiographic finding in at least one case in the last 20 years [5]. We herein report two autopsy cases of treated pleural mesothelioma in which marked lymphatic involvement was identified based on the radiologic-pathologic correlation.

## 2. Case Presentation

### 2.1. Clinical Summary

#### 2.1.1. Case 1

A 67-year-old male patient presented to us with a history of pleural effusion. He had a medical history of hypertension, cardiac arrhythmia, and acute hepatitis.

Prior to being referred to our institution for further treatment, he had been evaluated at another institute. A presumptive diagnosis of lung adenocarcinoma was made and he was treated with chemotherapy. A computed tomography (CT) scan showed right-sided pleural effusion with pleural thickening. Biopsies of the pericardium and pleura were performed, and the patient was diagnosed with epithelioid malignant mesothelioma. The patient had been regularly exposed to asbestos during 10 years of employment as a seaman. Because the patient did not wish to undergo surgery, he subsequently received three cycles of chemotherapy including cisplatinum and pemetrexed. Two cycles of chemotherapy with pemetrexed and carboplatin were administered; however, his condition did not improve.

**Figure 1:**
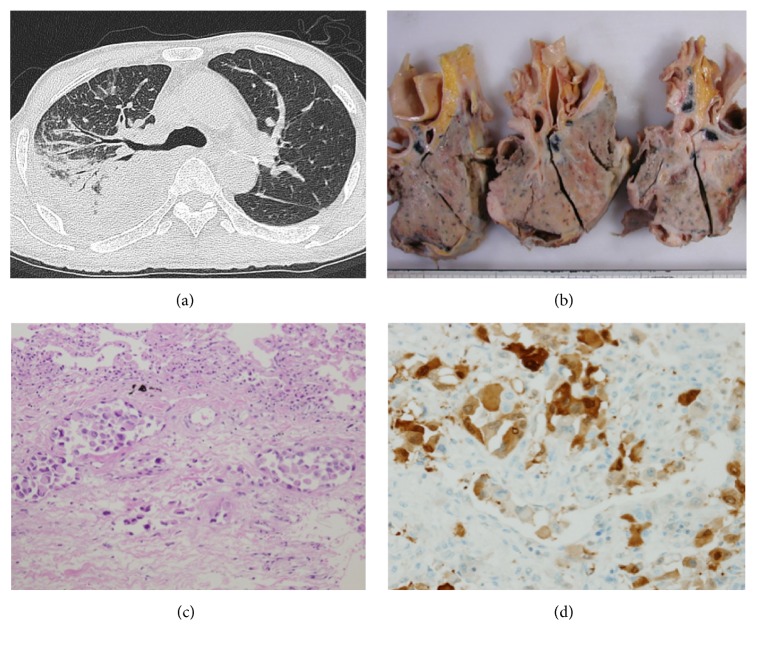
(a) An axial high-resolution chest CT scan showed extensive bronchovascular bundle and septal thickening and a consolidative mass in the upper lobe of the right lung; these findings are consistent with the lymphangitic spread of the tumor. (b) The gross pathological findings of the lung and pleura at autopsy showing multiple pleural nodules and masses on the pleural surface. The lung parenchyma is encased by tumor growth and the lymphatic spread of the tumor is observed. (c) Light microscopy of the resected pleural tumor. The lesion was histologically diagnosed as epithelioid-type malignant pleural mesothelioma. The marked invasion of the lymphatic vessel by tumor cells was observed (hematoxylin and eosin staining, ×100). (d) On immunohistochemical staining, the tumor cells were positive for calretinin, a mesothelial cell marker.

A CT scan showed marked right pleural effusion and mediastinal lymphadenopathy in addition to a consolidative mass of the upper lobe with bronchovascular bundle and septal thickening, suggesting lung parenchymal involvement and the lymphangitic spread of the tumor (Figure 1(a)). The bilateral pleural effusion increased in volume, and the patient ultimately died of respiratory failure at 16 months after the diagnosis.

#### 2.1.2. Case 2

A 68-year-old male presented with a cough and dyspnea approximately 4 months prior to his admission. She had no significant past medical history. A cytological examination performed in another institute suggested malignant pleural mesothelioma or adenocarcinoma. He was referred to our institution for further treatment. A CT scan showed left-sided pleural effusion and pleural thickening, some of which contained calcification. A histopathological examination of the pleura confirmed the diagnosis of epithelioid malignant mesothelioma. The patient had been regularly exposed to asbestos during 25 years of employment as a seaman.

**Figure 2:**
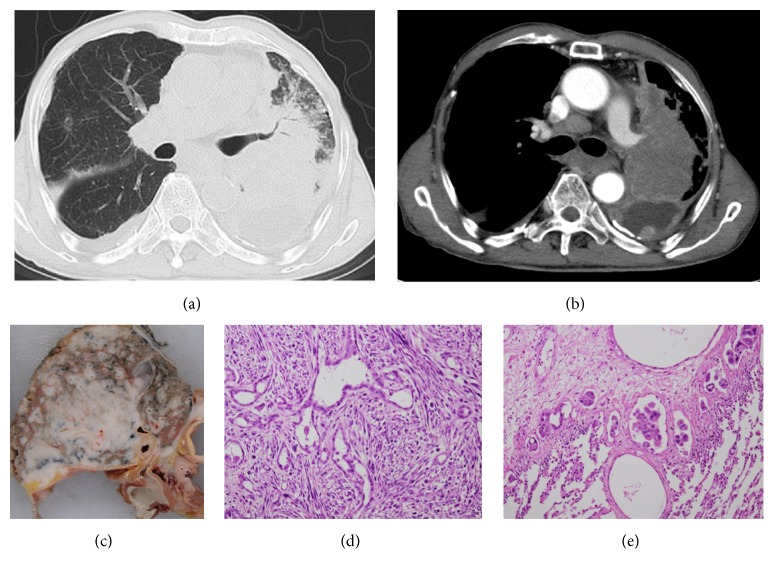
(a) An axial chest CT scan showed extensive bronchovascular bundle and septal thickening, an upper lobe consolidative mass, and perilymphatic nodules in the left lung; these findings are consistent with the lymphangitic spread of the tumor. (b) A contrast-enhanced chest CT scan showing a left upper lobe consolidative mass, left-sided pleural effusion, and pleural thickening with mediastinal lymph node swelling. (c) The gross pathological findings of the lung and pleura at autopsy. The lung parenchyma is encased by tumor growth and the lymphatic spread of the tumor is observed. (d) A low-power magnification view of the pleural tumor. The tumor is composed of epithelioid and spindle cells (hematoxylin and eosin staining, ×100). (e) Low-power magnification of the pleural tumor. The lesion was histologically diagnosed as biphasic-type malignant pleural mesothelioma. The photomicrograph shows invasion of the lymphatic channels by the tumor (hematoxylin and eosin staining, ×100).

A CT scan performed after four cycles of chemotherapy with the combination of cisplatinum and pemetrexed revealed left-sided pleural effusion, a consolidative mass with bronchovascular bundle, and septal thickening, suggesting lung parenchymal involvement and the lymphatic spread of the tumor (Figures 2(a) and 2(b)). Metastasis to the mediastinal lymph nodes with peritoneal metastasis and ascites were also present (Figure 2(b)). The patient ultimately died of respiratory failure at 18 months after the diagnosis.

### 2.2. Pathological Findings

#### 2.2.1. Case 1

At autopsy, the right whole lung showed severe adhesion to the right pleural cavity. The right parietal and visceral pleura had thickened and surrounded the right lung. The tumor was found to be primarily located in the right pleura invading the pulmonary parenchyma (Figure 1(b)). On the cut surface, the tumor was white in color and solid and firm in consistency. The tumor also invaded the peritoneal cavity, diaphragm, and trachea. Microscopically, the tumor cells showed a glandular pattern and were suggestive of malignant epithelioid mesothelioma. The tumor cells were epithelioid cells with eosinophilic cytoplasm infiltrating the pulmonary parenchyma with the lymphatic spread of the tumor (Figure 1(c)). An immunohistochemical examination was positive for calretinin and D2-40 and negative for TTF-1, SP-A, Ber-EP4, and carcinoembryonic antigen (CEA) (Figure 1(d)). Immunohistochemical markers are used to differentiate malignant mesothelioma from adenocarcinoma. Epithelioid-type malignant mesothelioma is positive for calretinin and D2-40 and negative for the TTF-1, SP-A, Ber-EP4, and CEA. Thus, the definite diagnosis was epithelioid-type malignant pleural mesothelioma.

#### 2.2.2. Case 2

At autopsy, the whole left lung showed severe adhesion to the left pleural cavity. The left parietal and visceral pleura had thickened and surrounded the left lung. The properties of the cut surface were similar to case 1. The tumor was found to be primarily located in the left pleura invading the lung parenchyma (Figure 2(c)). The tumor spread across the mediastinum and involved the opposite pleural cavity. The penetration of the diaphragm was also observed. Microscopically, the tumor cells were a biphasic lesion of malignant pleural mesothelioma (Figure 2(d)) and invaded the pulmonary parenchyma with the marked lymphatic spread of the tumor (Figure 2(e)). Immunohistochemically, the epithelioid tumor cells were positive for calretinin and D2-40 and AE1/AE3 and negative for TTF-1, Napsin A, Ber-EP4, and CEA. The spindle-shaped cells were also similar. Thus, the definite diagnosis was biphasic-type malignant pleural mesothelioma.

## 3. Discussion

Malignant mesothelioma is a rare neoplasm that arises from the pleura or, rarely, the pericardium or peritoneum. Microscopically, malignant mesothelioma is classified into 3 histologic subtypes that determine prognosis and therapy. These subtypes are epithelial, sarcomatoid, and mixed or biphasic. Malignant pleural mesothelioma, unlike lung cancer, progresses in a locally aggressive manner via invasion into the chest wall, mediastinum, and diaphragm. In the largest postmortem case series of patients with malignant plural mesothelioma, extrapleural dissemination was seen in 87.7% and extrathoracic metastasis in 55.4% [3]. Major sites for metastases are regional lymph nodes, lung, adrenal glands, liver, and kidneys.

Previous studies have reported that lymph node metastasis was significantly more common in epithelial and biphasic containing tumors than in sarcomatoid mesotheliomas. The rate of any extrapleural spread was high across all tumor types, with no statistically significant differences [3]. Lymphatic spread may occur in advanced cases of malignant pleural mesothelioma, such as the present cases, and has been well documented in histopathological studies. Nind et al. showed that the patterns of pulmonary parenchymal growth of malignant pleural mesothelioma were directly subpleural, lymphangitic, and other [4]. They described the histological patterns of lung parenchymal involvement with diffuse pleural mesothelioma. The lymphangitic involvement of the lung parenchyma was seen in 27 cases among a series of 200 cases of malignant pleural mesothelioma, including 16 epithelioid lesions, 3 biphasic lesions, and 8 sarcomatoid lesions. Our cases included 1 epithelioid type and 1 biphasic type.

Kadota et al. [6] correlated the histologic subtypes of epithelioid diffuse malignant pleural mesothelioma with the clinicopathological features. Five histological subtypes (tubulopapillary, trabecular, solid, micropapillary, and pleomorphic) were evaluated, and lymphatic invasion, which was detected in 44% of cases, was associated with the micropapillary (95%) and pleomorphic (68%) subtypes.

Although malignant lymphangiosis is known to occur as a microscopic phenomenon, as mentioned above, mesothelioma presenting as malignant lymphangiosis with a clear clinical and radiological picture is rare. To the best of our knowledge, the diffuse lymphatic spread of malignant pleural mesothelioma has been previously reported as the predominant radiographic finding in at least one case in the last 20 years [5]. Edde et al. [5] demonstrated a case of right pleural mesothelioma in which neoadjuvant chemotherapy was administered prior to right extrapleural pneumonectomy followed by adjuvant chemotherapy. After 21 months, CT showed diffuse parenchymal infiltration and lymphangitic spread over the contralateral lung. Similarly to our cases, this case resulted in a quick progression to respiratory failure and death.

Kim et al. analyzed multidetector CT images of 103 patients with malignant pleural mesothelioma and 24 patients with metastatic pleural disease from extrathoracic malignancy. They reported lymphangitic metastases were observed in 6 patients (5.8%) of the malignant pleural mesothelioma and 2 patients (8.3%) of the metastatic pleural disease [7]. In this way, the frequency of lymphatic involvement in malignant pleural mesothelioma differs between the histopathology and radiology.

In the present cases, CT showed a consolidative mass with bronchovascular bundle and septal thickening, mimicking lung cancer with lymphangitic carcinomatosis. The features of lymphangitic carcinomatosis are infiltration of cancer cells and interstitial edema in and around lymphatic vessels caused by lymph node metastasis in the lung. The metastatic cancer in the mediastinal and hilar lymph nodes may obstruct lymphatic drainage, resulting in retrograde spread of tumor into terminal lung tissues via lymphatic vessels. In addition, tumor microemboli may form in the terminal vessels of the lung due to hematogenous metastasis, which can invade the interstitium surrounding lymphatic vessels [8].

According to the TNM classification, ipsilateral parietal pleura is involved in early stage and later visceral pleura is involved [9]. The lymphatic drainage system of the pleura is complex and is important in the assessment of the spread of malignant mesothelioma. The visceral pleura drains to the same nodal groups as the lung parenchyma: bronchopulmonary, hilar, mediastinal, supraclavicular, and scalene. However, the parietal pleura has a different drainage into internal mammary, cardiophrenic, extrapleural, and intercostal nodes [10, 11].

Kadota et al. showed that lymphatic invasion showed a strong association with lymph node metastasis and an increased risk of death [6]. In the present cases, metastasis to the mediastinal lymph nodes was also present. The two cases were similar in that both patients had advanced-stage disease. In both cases, the pathological findings also showed the involvement of the lung parenchyma with the marked lymphatic spread of the tumor. We should keep this growth pattern in mind when treating patients with advanced-stage malignant pleural mesothelioma.

In conclusion, we reported two cases of malignant pleural mesothelioma with marked lung parenchymal and lymphatic involvement of the tumor. Although this growth pattern is unusual, malignant pleural mesothelioma should be considered as the differential diagnosis, especially in patients with pleural lesions.
